# The role of [^99m^Tc]Tc-HFAPi SPECT/CT in patients with malignancies of digestive system: first clinical experience

**DOI:** 10.1007/s00259-022-06068-1

**Published:** 2022-12-07

**Authors:** Xi Jia, Xinru Li, Bing Jia, Ye Yang, Yuanbo Wang, Yan Liu, Ting Ji, Xin Xie, Yu Yao, Guanglin Qiu, Huixing Deng, Zhaohui Zhu, Si Chen, Aimin Yang, Rui Gao

**Affiliations:** 1 Department of Nuclear Medicine, The First Affiliated Hospital of Xi’an Jiaotong University, Xi’an, 710061 People’s Republic of China; 2 Medical Isotopes Research Center and Department of Radiation Medicine, School of Basic Medical Sciences, Peking University, Beijing, 100191 People’s Republic of China; 3 Department of Medical Oncology, The First Affiliated Hospital of Xi’an Jiaotong University, Xi’an, 710061 People’s Republic of China; 4 Department of General Surgery, The First Affiliated Hospital of Xi’an Jiaotong University, Xi’an, 710061 People’s Republic of China; 5 Department of Nuclear Medicine, Peking Union Medical College Hospital, Chinese Academy of Medical Sciences and Peking Union Medical College, Beijing, 100730 People’s Republic of China; 6 Tianfu Technology Center, Foshan Atomical Medical Equipment Ltd.(S.C.), Foshan, 528000 People’s Republic of China

**Keywords:** [^99m^Tc]Tc-HFAPi, SPECT/CT, Digestive system cancer, Diagnostic efficiency

## Abstract

**Background:**

Recently, PET/CT imaging with radiolabelled FAP inhibitors (FAPIs) has been widely evaluated in diverse diseases. However, rare report has been published using SPECT/CT, a more available imaging method, with [^99m^Tc]Tc-labelled FAPI. In this study, we evaluated the potential effect of [^99m^Tc]Tc-HFAPi in clinical analysis for digestive system tumours.

**Methods:**

This is a single-centre prospective diagnostic efficiency study (Ethic approved No.: XJTU1AF2021LSK-021 of the First Affiliated Hospital of Xi’an Jiaotong University and ChiCTR2100048093 of the Chinese Clinical Trial Register). Forty patients with suspected or confirmed digestive system tumours underwent [^99m^Tc]Tc-HFAPi SPECT/CT between January and June 2021. For dynamic biodistribution and dosimetry estimation, whole-body planar scintigraphy was performed at 10, 30, 90, 150, and 240 min post-injection in four representative patients. Optimal acquisition time was considered in all the patients at 60–90 min post-injection, then quantified or semi-quantified using SUV_max_ and T/B ratio was done. The diagnostic performance of [^99m^Tc]Tc-HFAPi was calculated and compared with those of contrast-enhanced CT (ceCT) using McNemar test, and the changes of tumour stage and oncologic management were recorded.

**Results:**

Physiological distribution of [^99m^Tc]Tc-HFAPi was observed in the liver, pancreas, gallbladder, and to a lesser extent in the kidneys, spleen and thyroid. Totally, 40 patients with 115 lesions were analysed. The diagnostic sensitivity of [^99m^Tc]Tc-HFAPi for non-operative primary lesions was similar to that of ceCT (94.29% [33/35] vs 100% [35/35], respectively; *P* = 0.5); in local relapse detection, [^99m^Tc]Tc-HFAPi was successfully detected in 100% (*n* = 3) of patients. In the diagnosis of suspected metastatic lesions, [^99m^Tc]Tc-HFAPi exhibited higher sensitivity (89.66% [26/29] vs 68.97% [20/29], respectively, *P* = 0.03) and specificity (97.9% [47/48] vs 85.4% [41/48], respectively,* P* = 0.03) than ceCT, especially with 100% (24/24) specificity in the diagnosis of liver metastases, resulting in 20.0% (8/40) changes in TNM stage and 15.0% (6/40) changes in oncologic management.

**Conclusion:**

[^99m^Tc]Tc-HFAPi demonstrates a greater diagnostic efficiency than ceCT in the detection of distant metastasis, especially in identifying liver metastases.

**Supplementary Information:**

The online version contains supplementary material available at 10.1007/s00259-022-06068-1.

## Introduction

An in-depth understanding of the tumour microenvironment has revealed a new player: cancer-associated fibroblasts (CAFs) [1]. The majority of epithelial tumours recruit fibroblasts and other non-malignant cells, stimulating them to become CAFs. This often leads to overexpression of membrane serine protease fibroblast activating protein alpha (FAP-α, also known as prolyl endopeptidase FAP), which is estimated to be overexpressed in approximately 90% of human cancers [2–4]. As FAP is mostly absent in healthy tissue, inhibitors of FAP (FAPIs) can be used in nuclear medicine for imaging [5]. Indeed, a large number of FAPI-based radiopharmaceuticals have been developed for PET/CT imaging, and a promising role for [^68^Ga]Ga-FAPI PET/CT in the diagnosis, staging, and radiotherapy planning of digestive tract cancers has been demonstrated [6–8].

[^68^Ga]Ga-FAPI PET/CT showed a higher sensitivity than [^18^F]FDG PET/CT in the detection of primary and metastatic lesions of various types of cancers [6, 9]. Recently, Koerber et al. reported the first clinical use of [^68^Ga]Ga-FAPI PET/CT for tumours in the lower intestinal tract [7]. Their results revealed that both primary and metastatic malignancies in the lower gastrointestinal tract can be reliably detected using [^68^Ga]Ga-FAPI PET/CT, leading to relevant changes in TNM status and oncologic management.

Due to its lower cost, SPECT/CT with technetium-99m (^99m^Tc) is a more widely available, and ^99m^Tc-labelled FAPIs are generally applicable tracers that are attractive options for imaging in clinical management when PET imaging is inaccessible or limited [10]. Lindner et al. reported ^99m^Tc-labelled FAPIs and evaluated their biodistribution in tumour‐bearing mice [10], and found ^99m^Tc-FAPI-34 showing strong and constant tumour accumulation. The preclinical application has indicated that it is a good candidate for scintigraphic imaging owing to the high contrast obtained via rapid tumour uptake and clearance from the rest of the body. Nevertheless, reliable clinical data are lacking, which only applied in two patients with ovarian and pancreatic cancer. Here, we report our first clinical experience with [^99m^Tc]Tc-HYNIC-FAPI-04 ([^99m^Tc]Tc-HFAPi) SPECT/CT applied in a cohort of patients with digestive system tumours. After quantifying tracer uptake in primary tumours and metastases, we compared the diagnostic efficiency of the [^99m^Tc]Tc-HFAPi with the conventional imaging ceCT, which is routinely recommended in digestive system tumours [11–13].

## Materials and methods

### Radiopharmaceutical preparation

For ^99m^Tc radiolabelling, 1 mL of 925–1295 MBq (25–35 mCi) of [^99m^Tc]TcO4^−^ saline solution was added to 25 µg of hydrazinonicotinamide-FAPI-04 (HYNIC-FAPI-04, abbreviated to HFAPi, Fig. S1), 3.0 mg of Trisodium triphenylphosphine-3,3′,3″-trisulfonate, and 2.0 mg of tricine, then incubated at 100 °C for 15 min. The radiochemical purity (RCP) was analysed by radio-HPLC and ITLC-SG, and the specific operation method is detailed in the supplementary information. For clinical use, the RCP was always greater than 95%. The reaction mixture was then filtered through a 0.20-mm Millex-LG filter (EMD Millipore) before agent injection.

### Patients

This is a single-centre prospective diagnostic efficiency study of [^99m^Tc]Tc-HFAPi SPECT/CT in digestive system tumours, with ceCT serving as the reference method, approved by the Clinical Research Ethics Committee of the First Affiliated Hospital of Xi’an Jiaotong University (Ethic approved No.: XJTU1AF2021LSK-021) and Chinese Clinical Trial Register (Registration No.: ChiCTR2100048093). From January to June 2021, patients with suspected digestive system tumours who needed preoperative initial staging or posttreatment restaging were consecutively recruited at the First Affiliated Hospital of Xi’an Jiaotong University with written informed consent. Detailed Eligibility criteria is provided in the supplementary information. After a standard work-up including but not limited to ceCT, additional [^99m^Tc]Tc-HFAPi SPECT/CT was performed (generally within 7 days).

### Scintigraphy and SPECT/CT

Based on the previous reference dose [10, 14, 15], [^99m^Tc]Tc-HFAPi was administered intravenously in amounts ranging from 790.4 to 930.2 MBq (21.36 to 25.14 mCi). For dynamic biodistribution, whole-body planar scintigraphy was performed at 10, 30, 90, 150 and 240 min in four representative patients. Base on biodistribution results, [^99m^Tc]Tc-HFAPi imaging was performed 60–90 min following tracer injection in all the patients to get whole-body planar scintigraphy as well as SPECT/CT tomography fusion images. Whole-body scans were performed via GE Discovery 670 pro scanner system (GE Healthcare) equipped with low-energy high-resolution (LEHR) collimators in 18 cm/min velocity. Low-dose CT was performed for attenuation correction and anatomic localization. The patients were asked to self-report any abnormalities at 30 min after the examination was completed.

### Biodistribution and dosimetry estimation

Visual analysis was applied to determine the integral biodistribution of the tracer as well as the transient and intersubject stability. For each subject, regions of interest (ROIs) were delineated over the identified organs: the heart, liver, lungs, kidneys, pancreas, spleen, brain, thyroid and salivary glands. The geometric mean count was determined for every organ from the background-corrected anterior and posterior counts. The results are expressed as a percentage of the initial injected activity after decay correction (%ID/organ). For dosimetry estimation, absorbance dose of different organs and effective dose were calculated using OLINDA/EXM 1.0 software (Vanderbilt, University, Nashville, TN, USA) as previously described [15].

### SPECT/CT imaging review

[^99m^Tc]Tc-HFAPi SPECT/CT scans were evaluated by 1 certified radiologist and 2 certified nuclear medicine physicians. They reach consensus when there is disagreement. Conventional imaging was interpreted by 2 certified radiologists with consensus but blind to the [^99m^Tc]Tc-HFAPi SPECT/CT results. Fused SPECT/CT images were viewed on the Xeleris Workstation (version AW 4.7, GE Healthcare). For quantitative and semi-quantitative analyses, ROIs were drawn on transaxial images over the tumour with focally increased uptake. Quantitative calculating the SUV_max_ was based on an algorithm, which has been patented (Patent number: US11189374B2) [16]. The tumour-to-background (T/B) ratio was determined by dividing the maximum tumour uptake by the maximum contralateral muscle uptake. For skull lesions, T/B ratio was calculated as dividing the maximum tumour uptake by the maximum normal skull uptake.

### Diagnosis and follow-up

Histopathology of biopsy/resected surgical specimens served as the gold standard for the final diagnosis. In cases in which the diagnosis of malignancy was not applicable, follow-up data after the SPECT/CT scans were requested. Referring to a similar study [9], the disease was defined as malignancy when (a) typical malignant features were confirmed by multi-modality imaging, (b) significant progression on follow-up imaging (significant increase in size), or (c) a significant decrease in size after anticancer treatment. All suspected lesions were followed up for no less than 6 months.

### Immunohistochemistry (IHC) of FAP expression

FAP expression in 4 representative patients was analysed by immunohistochemistry, heat-mediated antigen retrieval was performed with Tris/EDTA buffer pH 9.0. The sections were incubated with 1:250 humanized anti-fibroblast activation antibody (Abcam, ab207178) at 4 °C overnight. After incubation with the labelled streptavidin–biotin (LSAB) complex, the slides were stained and visualized using the iView DAB detection system (ZSGB-BIO, Beijing, China). Typical lesions in high-power fields were photographed for visual comparison.

### Statistical analyses

All statistical analyses were conducted using SPSS 25.0 statistical analysis software (IBM, Armonk, NY, USA). For organ biodistribution, the percentage of initial injected activity after decay correction (%ID) was used. To determine lesion uptake, the T/B ratio and SUV_max_ were used with the median ± interquartile range (IQR) because of non-normal distribution. McNemar test and chi-square test were employed to compare the diagnostic values between [^99m^Tc]Tc-HFAPi SPECT/CT and ceCT. A receiver operating characteristic (ROC) curve was constructed to quantify the diagnostic performance of the T/B ratio and SUV_max_ by assessing the respective areas under the curve (AUCs). Two-tailed *P* values < 0.05 were considered significant.

## Results

### Radiopharmaceutical preparation

The structure of [^99m^Tc]Tc-HFAPi was shown in (Fig. S1). The average radiochemical purity of [^99m^Tc]Tc-HFAPi prepared from lyophilized kits, determined by radio-HPLC (Fig. S2) and ITLC-SG (Fig. S3), was over 95% with < 1% of free [^99m^Tc]TcO_4_^−^ as well as < 0.5% of [^99m^Tc]Tc-colloid. [^99m^Tc]Tc-HFAPi could be readily prepared in high specific activity (> 2.275 × 10^5^ MBq/μmoL), and it was stable in the kit matrix as well as in the saline for > 6 h. More data on [^99m^Tc]Tc-HFAPi preparation and preclinical studies will be reported in detail in a separate research paper.

### Patient characteristics

**Fig. 1:**
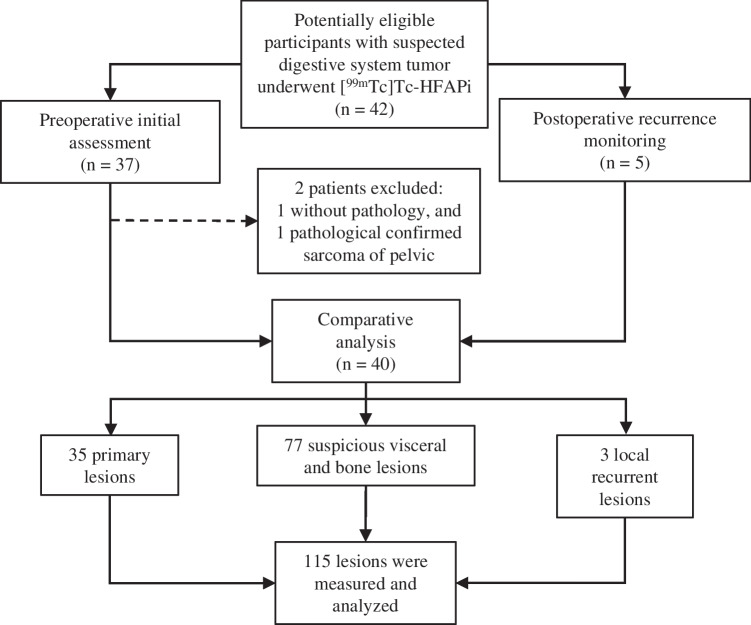
The scheme of the study design

For 40 patients (25 male) enrolled, thirty-five patients had yet to undergo cancer-related surgery (30 treatment naïve and 5 with chemo/radio-therapy). Another 5 patients had already undergone surgery with/without chemotherapy and/or radiotherapy. The characteristics of the patients and primary lesions information are summarized in Table 1. Ultimately, 39 patients were confirmed to have malignant disease, whereas 1 was confirmed to have benign tumour (spindle cell tumour of intestine). The scheme of the study design is presented in Fig. 1.

**Table 1:** Characteristics of patients that underwent [^99m^Tc]Tc-HFAPi SPECT/CT

Characteristic	Value
No. of patients	40
Age (years)	
Median	64.5
Interquartile range	54.3–71.8
Sex	
Male	25
Female	15
Site and pathology of primary disease	
Rectum Adenocarcinoma	15
Gastric Adenoca**r**cinoma	13
Colonic Adenocarcinoma	8
Esophageal Squamous carcinoma	1
Intestinal Spindle cell tumour	1
Pancreatic carcinoma	1
Anal malignant melanoma	1
Clinical status before imaging	
Treatment-naive	30
Neoadjuvant chemo/radio-therapy	5
Resection surgery	3
Chemo/radio-therapy after surgery	2
Other imaging	
Contrast enhanced CT	40
Gastrointestinal endoscope	38
DWI	11
B ultrasound	7
^18^F-FDG	1
Clinical questions for ^99m^Tc-HFAPi	
Staging of cancer before surgery	35
Identification of disease recurrence and restaging	5

No drug-related side effects occurred during or after [^99m^Tc]Tc-HFAPi injection, and SPECT/CT imaging was tolerated well by all patients. Vital parameters remained stable, and no patient reported any new symptoms during the observation period.

### Dynamic biodistribution in organs and dosimetry estimation

**Fig. 2:**
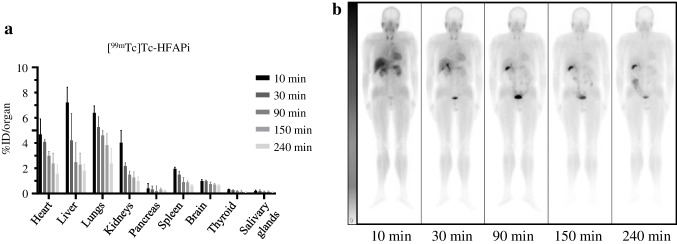
Biodistribution of [^99m^Tc]Tc-HFAPi in different vital organs over time. **a** % Injection Dose (ID)/organ of [^99m^Tc]Tc-HFAPi in heart, liver, lungs, kidneys, pancreas, spleen, brain, thyroid and salivary glands in different times. Data represent median ± interquartile range. **b** Example of background ROIs in patient #002 on planar scintigraphy images: whole body [^99m^Tc]Tc-HFAPi scintigraphy was performed at 10, 30, 90, 150 and 240 min post-injection

The dynamic physiological biodistribution of [^99m^Tc]Tc-HFAPi in vital organs at 10, 30, 90, 150, and 240 min was measured in 4 patients and summarized using %ID/organ (patients’ information Table S1). Physiological distribution of [^99m^Tc]Tc-HFAPi was observed in the liver, pancreas, gallbladder, and to a lesser extent in the kidneys, lungs, spleen, salivary glands, and thyroid glands, with rapid clearance of the radiotracer from these organs (Fig. 2a). Representations of a coronal section from whole-body SPECT are shown in Fig. 2b.

A summary of dosimetric parameters for various organs is given in Table S2, and the mean effective dose equivalent of the whole body was 1.26 × 10^−3^ mSv/MBq, which is consistent with those for other molecules labelled with ^99m^Tc [15, 17].

### [^99m^Tc]Tc-HFAPi for diagnosing primary lesions

**Fig. 3:**
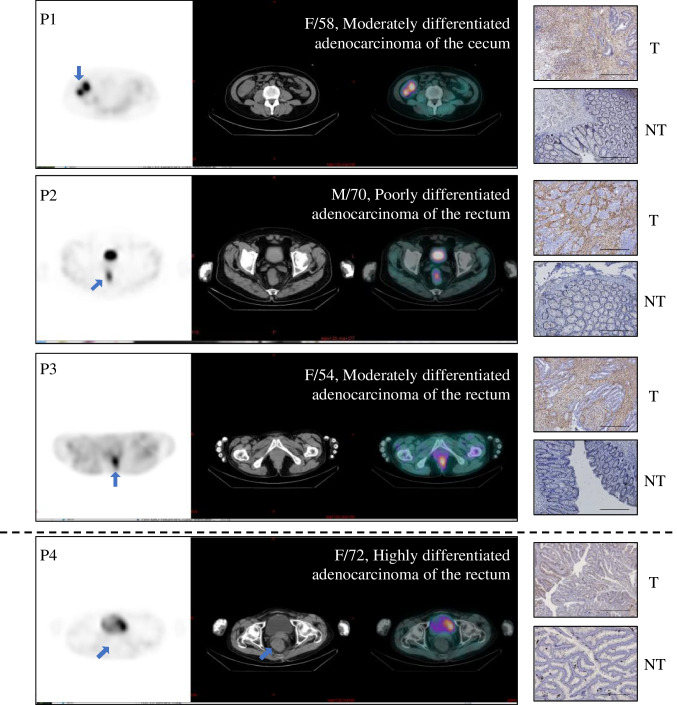
SPECT/CT (left) and immunohistochemistry staining (right) of representative primary lesions and tumour-adjacent tissue. The graphs above the dotted line were representations of true positive cases (P1–P3), while below the dotted line is a representation of false negative case (P4) by [^99m^Tc]Tc-HFAPi. Scale bar, 200 μm; T: primary tumour; NT: Tumour-adjacent tissue

Thirty-five patients with unresected primary digestive system lesions, which were all pathologically confirmed as malignant lesions, were detected by [^99m^Tc]Tc-HFAPi SPECT/CT with a nearly identical sensitivity of 94.29% (33/35) as ceCT (35/35, 100%, *P* = 0.5). The two false-negative of [^99m^Tc]Tc-HFAPi were found highly differentiated rectal adenocarcinoma by pathology. Representative 3 true-positive and 1 false-negative lesions were stained with the anti-FAP antibody by IHC. As illustrated in Fig. 3, patient (P4) with false-negative lesions on [^99m^Tc]Tc-HFAPi SPECT/CT showed the lowest expression of FAP compared with true-positive patients (P1-3), indicating that the uptake of [^99m^Tc]Tc-HFAPi was associated with the expression of FAP.

Local recurrence was found in 60% (3/5) of patients by pathology or follow-up imaging, all of which were positively detected by ceCT and [^99m^Tc]Tc-HFAPi SPECT/CT **(**Table S3).

### [^99m^Tc]Tc-HFAPi in the diagnosis of suspected metastatic lesions

After [^99m^Tc]Tc-HFAPi examination, 77 suspected lesions were detected. Among them, 29 lesions in 9 patents were confirmed metastatic lesions by pathology (*n* = 4), multi-modality imaging (*n* = 11), or follow-up (*n* = 14). In total, [^99m^Tc]Tc-HFAPi-positive metastases were observed in 8 patients with 26 lesions, including liver (*n* = 15), bone (*n* = 8), abdomen (*n* = 1), pelvic (*n* = 1) and mediastinum tissue (*n* = 1). Compared to ceCT, [^99m^Tc]Tc-HFAPi exhibited higher sensitivity in the diagnosis of suspected metastatic lesions (89.66% [26/29] vs 68.97% [20/29], respectively, *P* = 0.03). For 48 benign lesions, [^99m^Tc]Tc-HFAPi showed higher specificity than ceCT (97.9% [47/48] vs. 85.4% [41/48], respectively; *P* = 0.03). The only one false positive case turned out to be tuberculosis that has been reported previously [9, 18]. A comparison of diagnostic efficiency on benign or metastatic lesions between ceCT and [^99m^Tc]Tc-HFAPi SPECT/CT is shown in Table 2. It is worth noting that for liver metastasis determination, 100% (24/24) specificity was achieved using [^99m^Tc]Tc-HFAPi SPECT/CT, with 83.3% (20/24) by ceCT (Table 3). Although the non-statistical difference (*P* = 0.13) might be related to the relatively small sample size, subsequent studies with larger sample size are still worthwhile.

**Table 2:** Diagnostic efficiency of [^99m^Tc]Tc-HFAPi in suspected lesions compared with ceCT

Basis of analysis and modality	Sensitivity TPR^a^ (%)	Specificity TNR^b^ (%)	Negative predict value NPV (%)	Positive predict value PPV (%)	Accuracy ACC (%)
HFAPi-meta 95% CI	89.7 (26/29)	97.9 (47/48)	94.0 (47/50)	96.3 (26/27)	94.8 (73/77)
	71.5–97.3	87.5–99.9	82.5–98.4	79.1–99.8	87.0–98.4
ceCT-meta 95% CI	69.0 (20/29)	85.4 (41/48)	82.0 (41/50)	74.1 (20/27)	79.2 (61/77)
	49.0–84.0	71.6–93.5	68.1–90.9	53.4–88.1	68.8–86.9

^a^*TPR*, true positive rate

^b^*TNR*, true negative rate

**Table 3:** Diagnostic efficiency of [^99m^Tc]Tc-HFAPi in liver metastasis compared with ceCT

Basis of analysis and modality	Sensitivity TPR (%)	Specificity TNR (%)	Negative predict value NPV (%)	Positive predict value PPV (%)	Accuracy ACC (%)
HFAPi-liver 95% CI	88.2 (15/17)	100.0 (24/24)	92.3 (24/26)	100.0 (15/15)	95.1 (39/41)
	62.2–97.9	82.8–100.0	73.4–98.7	74.7–100.0	83.0–99.5
ceCT-liver 95% CI	100.0 (17/17)	83.3 (20/24)	100.0 (20/20)	81.0 (17/21)	90.2 (37/41)
	77.1–100.0	61.8–94.5	79.9–100.0	57.4–98.7	76.9–96.7

### Clinical values of [^99m^Tc]Tc-HFAPi

In suspected metastatic lesions which were not diagnosed coincidently by ceCT and [^99m^Tc]Tc-HFAPi SPECT/CT, follow-up data were requested as described in methods (Diagnosis and follow-up section). As a result, [^99m^Tc]Tc-HFAPi SPECT/CT suggested the metastasis (M) classification restaging in 8/40 (20.0%) patients (1 patient staged up and 7 staged down). Among the restaging, 6 of 8 patients changed the oncologic regimen, including 1 with new findings for bone metastasis who changed to systemic therapy and 5 for whom curative surgery was performed instead of systemic therapy, consistently with those of [^99m^Tc]Tc-HFAPi imaging results. Changes in the clinical oncologic regimen are given in Table 4. The results indicate that [^99m^Tc]Tc-HFAPi SPECT/CT can provide a strong basis for clinical decision-making.

**Table 4:** Changes in metastatic staging and oncologic management according to [^99m^Tc]Tc-HFAPi

No	Sex	Age	State	Primary tumour	Metastasis stage and site from ceCT	Metastasis stage and site from [^99m^Tc]Tc-HFAPi	Ways to confirm	Changing in stage	Clinical decision
1	F	69	Treatment-naive	Adenocarcinoma of rectum	Mx	M1 with occipital bone	CT with bony change	Staging up	Palliative operation
2	F	58	Treatment-naive	Adenocarcinoma of the cecum	M1 with liver	M0 without liver	DWI: hepatic cyst	Staging down	Tend to operation
3	M	81	Treatment-naive	Adenocarcinoma of rectum	M1 with liver	M0 without liver	DWI: Hepatic spongy hemangioma	Staging down	Tend to operation
4	M	55	Treatment-naive	Gastric adenocarcinoma	M1 with liver	M0 without liver	DWI: no obvious abnormality	Staging down	Tend to operation
5	F	22	Treatment-naive	Gastric adenocarcinoma	M0	M1 with skull, posterior bulbar tissue, peritoneum	Pathology of peritoneum, CT with bony change of the skull	Staging up	Palliative surgery and intraperitoneal chemotherapy
6	M	58	Treatment-naive	Gastric adenocarcinoma	Mx	M0 without liver	Follow up	Staging down	Tend to operation
7	F	51	Neoadjuvant chemotherapy	Adenocarcinoma of rectum	Mx	M0 without spleen	DWI: Splenic hemangioma	Staging down	Tend to operation

### SUV_max_ and T/B ratio in the diagnosis of benign and malignant disease

**Fig. 4:**
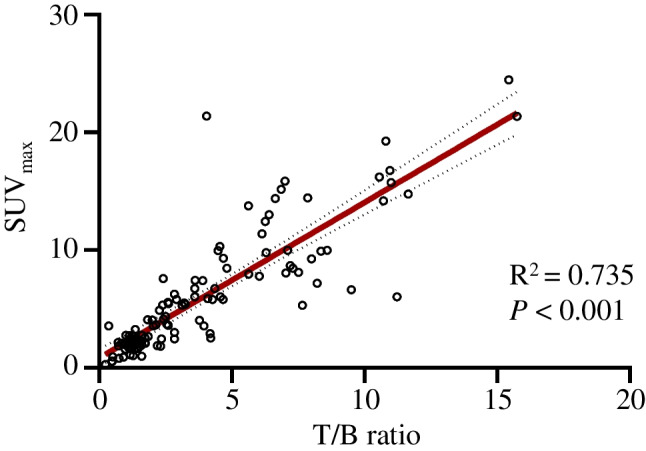
Linear relationship between SUV_max_ and T/B ratio. R: linear correlation coefficient

Our team previously designed an algorithm, which has been patented (Patent number: US11189374B2) [16], to calculate the SUV_max_ based on SPECT/CT and linearized it against the standard T/B ratio. The result indicating an obvious linear correlation with *R*^2^ of 0.735 (Fig. 4, *P* < 0.001) between SUV_max_ and T/B ratio.

**Fig. 5:**
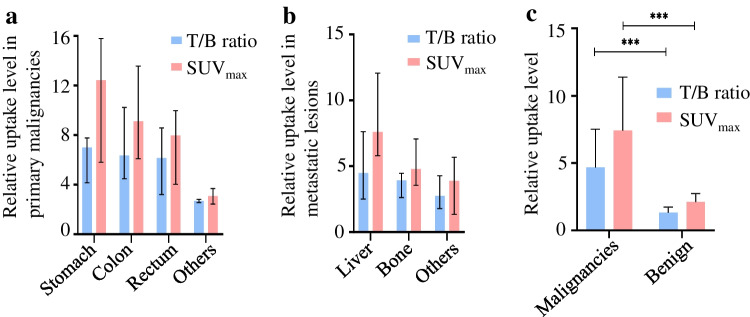
Uptake of [^99m^Tc]Tc-HFAPi in different lesions. T/B ratio (blue bar) and SUV_max_ (red bar) are shown in primary malignancies (**a**) and metastatic lesions (**b**). **c** The comparison of [^99m^Tc]Tc-HFAPi uptake between benign and malignant lesions. Data represent median ± interquartile range. ***, *P* < 0.001

For primary malignant lesions, the median T/B ratio and SUV_max_ were 6.35 (IQR: 3.64 to 8.10) and 9.52 (IQR: 5.73 to 14.52), respectively; for all metastases, they were 3.65 (IQR:2.47 to 4.79) and 6.05 (IQR: 4.43 to 9.09), respectively. The SUV_max_ and T/B ratio for different malignant lesions by [^99m^Tc]Tc-HFAPi are shown in Fig. 5a, b and Table S3, S4. High SUV_max_ and T/B ratios were found in gastric cancer and liver metastasis. For non-malignant lesions, few showed intense uptake of [^99m^Tc]Tc-HFAPi, with a median T/B ratio and SUV_max_ of 1.32 (IQR: 1.01–1.74) and 2.11 (IQR: 1.68–2.75), respectively, both were significantly lower (*P* < 0.001) than malignancies (Fig. 5c).

**Fig. 6:**
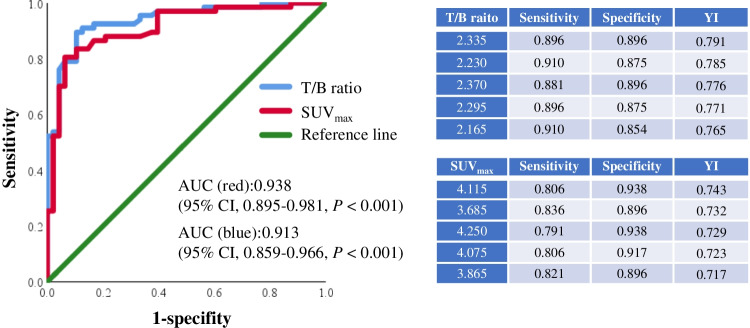
The performance of T/B ratio (red line) and SUV_max_ (blue line) in the diagnosis of digestive system cancer by receiver operating characteristic (ROC) curves analysis (left). The charts (right) show several optimal cut-off values for discriminating malignant from non-malignant lesions. T/B ratio, tumour/background ratio; AUC, area under ROC curves; YI, Youden’s index

The ROC curve built with 67 malignant lesions (38 primary malignancies and 29 metastatic lesions) and 48 benign lesions yielded an AUC of 0.938 (95% CI, 0.895–0.981, *P* < 0.001) for the T/B ratio and 0.913 (95% CI, 0.859–0.966, *P* < 0.001) for SUV_max_ (Fig. 6, left). Youden’s index analysis revealed several optimal cut-off values for discriminating malignant from non-malignant lesions (Fig. 6, right).

## Discussion

Recent PET/CT studies with FAP inhibitors have been well developed, revealing strong PET signals across dozens of major cancers, especially digestive system cancers [6, 7, 19, 20]. With FAPI-specific PET imaging, patients do not require dietary preparation, and high-quality images can be obtained soon after tracer injection (10 min to 1 h, mainly 1 h) [21]. The superior value of [^68^Ga]Ga-FAPI PET/CT over [^18^F]FDG in detecting primary and metastatic lesions in digestive system cancers has been confirmed, most lesions showing higher tracer uptake with FAPI imaging, and it exhibits a promising role in the diagnosis, (re)staging, management, and treatment planning of digestive system cancers [22]. However, limited data about ^99m^Tc-labelled FAPIs in clinical have been reported, with only application in two patients (ovarian and pancreatic cancer) [10]. Here, we evaluated the biodistribution of a newly developed [^99m^Tc]Tc-HFAPi and the uptake in different digestive system cancers. Biodistribution studies have shown that [^99m^Tc]Tc-HFAPi has good targeting of malignancies and fast renal clearance, with low uptake in normal organs, leading to a higher tumour-to-background ratio and have a relatively wide imaging time window, indicating it is a suitable clinical imaging agent for digestive system tumours. Comparisons of diagnostic efficiency in digestive system cancers between [^99m^Tc]Tc-HFAPi and ceCT were performed in this study, since CT with contrast is routinely used for preoperational imaging in digestive system cancers based on NCCN guidelines [11–13].

Our study demonstrated that [^99m^Tc]Tc-HFAPi and ceCT were comparable in detecting the primary tumours of digestive system. Almost all primary malignancies (*n* = 33) showed marked uptake of [^99m^Tc]Tc-HFAPi, especially gastric cancers and colon cancers, with median T/B ratios of 7.01 and 6.35, and median SUV_max_ values of 12.43 and 9.13, respectively. This was in line with the previous PET imaging with ^68^Ga-labelled FAPI-04 [20], which showed the highest uptake in colon cancers and gastric cancers. Our study also covered small samples of oesophageal squamous carcinoma, pancreatic carcinoma, and anal malignant melanoma, which all showed good detection performance. In the detection of tumour recurrence in patients who received surgery (n = 5), 60% (3/5) of them showed recurrence and all had successful detection by [^99m^Tc]Tc-HFAPi, consistent with the ceCT results. Although the samples were too few, they were particularly useful for their presence of elevated tumour markers but no clinical or morphological evidence, as previously indicated [9].

The liver is the main site of metastasis and a major cause of death in digestive system malignancies, leading to short PFS and extremely poor prognosis [23]. Several imaging methods have been utilized in the detection of liver metastasis, including ceCT, MRI, and [^18^F]FDG PET/CT, but all have limitations. Although ceCT is commonly used for diagnosing liver metastasis, its accuracy does not always meet clinical requirements [24, 25]. ceMRI is suggested to have advantage over ceCT in detecting small liver metastases (< 10 mm); however, criticism has been directed towards it because the cost does not match the clinical benefit [24, 26, 27]. [^18^F]FDG PET/CT is not routinely indicated for initial staging of digestive tract tumours [11–13] because of its low sensitivity for liver metastasis, particularly in patients who have received preoperative chemotherapy [25, 28, 29]. The low background of the normal liver leads to the potential application of FAPI tracers in patients with suspected liver metastases [7, 30]. Previous studies with PET FAPI agents have indicated an outstanding role in the diagnosis of liver metastasis [7, 20]. Our findings likewise support the implementation of [^99m^Tc]Tc-HFAPi SPECT/CT in the identification of liver metastasis. In metastatic lesions, the highest uptake of [^99m^Tc]Tc-HFAPi were achieved in liver metastasis, with a median T/B ratio and SUV_max_ of 4.48 and 7.59, respectively. Basically, [^99m^Tc]Tc-HFAPi demonstrated satisfactory sensitivity (88.2%) for detecting liver metastasis. Moreover, due to the low expression of FAP in benign liver lesions [31], an extremely high specificity (100%) of [^99m^Tc]Tc-HFAPi was achieved. Four cases of suspected liver metastasis in ceCT were negatively detected by [^99m^Tc]Tc-HFAPi with minimal uptake, and the lesions were proven to be benign by biopsy or multi-modality imaging, thus excluding from metastasis and restaging from M1 to M0 and allowing the chance for radical surgery. Overall, [^99m^Tc]Tc-HFAPi provided an accurate diagnosis of suspected liver metastases, which may avoid unnecessary misdiagnosis, correct tumour staging and promote clinical oncological decisions.

In addition to liver metastasis, [^68^Ga]Ga-FAPI outperformed traditional imaging in detecting bone metastases [9]. In the present study, 2 patients with multiple skeletal metastases showed visible uptake of [^99m^Tc]Tc-HFAPi, which was often missed by ceCT. These results further demonstrate the diagnostic advantage of [^99m^Tc]Tc-HFAPi for various distant metastasis, thus improving the staging of cancer and treatment modification.

The expression of FAP is also widely reported to be avid in tissue modelling, wound healing, and inflammation-induced fibrosis [32]. Our study with ^99m^Tc-labelled FAPI also demonstrated pulmonary tuberculosis with moderate uptake (T/B ratio of 4.36). Moreover, uterine fibroids demonstrated diffuse uptake of [^99m^Tc]Tc-HFAPi, which might be attributed to the activated fibroblasts, as shown in previous PET imaging studies [33, 34].

Altogether 2 primary tumour lesions demonstrated false-negative uptake of [^99m^Tc]Tc-HFAPi. One might be attributed to obviously low expression of FAP, which was revealed by following IHC staining [35], while the other one still had moderate expression on IHC. The mechanisms underlying the discordance between FAP expression and [^99m^Tc]Tc-HFAPi uptake might be due to the liganding of molecules and certain physical influences, which might alter the conformation of some membrane proteins and their functional state (activation or inactivation) [36]. When it is in a nonactivated conformation, it is inaccessible to its targeted inhibitors. However, this still needs to be further confirmed.

We know that sensitivity and specificity are equally important when Youden’s index is used to obtain the best cut-off value. Previous study adopted cut-off value based on the highest Youden’s index [37]. However, a fixed cut-off value often does not meet the needs of clinical decision-making. Different cut-off values are needed according to different disease states and clinical purposes, just as previous study indicated [38]. Here, we listed a series of cut-off values for reference, because larger samples and data from other tumour histotypes and different clinical statuses with [^99m^Tc]Tc-HFAPi are needed to optimize this criterion.

There are several limitations to this study. First, a small patient cohort limited the statistical significance for some kinds of cancers, such as oesophageal, pancreatic, and gallbladder cancer. Second, although being the ideal reference standard, histopathological examination was not available in all lesions because of ethical and technical reasons. Third, further prospective studies with larger populations in head-to-head comparisons of [^99m^Tc]Tc-HFAPi SPECT/CT and [^68^Ga]Ga-FAPI PET/CT are warranted to best comment on the superiority of the tracers to clarify the role of SPECT/CT.

Despite these limitations, to the best of our knowledge, this article might be the first application of a new ^99m^Tc-labelled FAPI for digestive system tumours from a clinical perspective, and we confirmed its diagnostic efficacy in tumour staging and restaging, providing an important basis for clinical application and subsequent studies. Furthermore, our ^99m^Tc-labelled FAPI might provide some future directions for drug labelling with ^188^Re, such as integration in FAP targeted diagnosis and targeted radionuclide therapy [10].

## Conclusion

In this work, we have developed a new ^99m^Tc-labelled molecular probe and transformed it for the first time for digestive system tumours study. The findings indicate selective uptake of [^99m^Tc]Tc-HFAPi SPECT/CT and demonstrate a high target-to-background ratio for various types of digestive system cancers as well as related metastasis, especially liver metastasis, which contributes to the current literature on FAP inhibitor molecular imaging. Further studies with large populations and other cancer types should be done to draw firmer conclusions on the superiority of the tracers.

## Supplementary Information

Below is the link to the electronic supplementary material.

Supplementary file1 (DOCX 119 KB)

## Data Availability

The datasets used or analyzed during the current study are available from the corresponding authors on reasonable request.
