# Supplementary material for: The role of [99mTc]Tc-HFAPi SPECT/CT in patients with malignancies of digestive system: first clinical experience
Source: Eur J Nucl Med Mol Imaging. 2022 Dec 7;50(4):1228–39. doi: 10.1007/s00259-022-06068-1 (PMC9931852; doi:10.1007/s00259-022-06068-1)
Supplement: Supplementary file 1 — Supplementary file1 (DOCX 119 KB) [file 259_2022_6068_MOESM1_ESM.docx]

**Supplementary Informations for**

**The role of [^99m^Tc]Tc-HFAPi SPECT/CT in patients with malignancies of digestive system: first clinical experience**

Xi Jia^1,#^ · Xinru Li^1,#^ ·Bing Jia^2,#^ · Ye Yang^1^ · Yuanbo Wang^1^ · Yan Liu^1^ · Ting Ji^1^ · Xin Xie^1^ · Yu Yao^3^ · Guanglin Qiu^4^ · Huixing Deng^1^ · Zhaohui Zhu^5^ · Si Chen^6^ · Aimin Yang^1^ · Rui Gao^1,*^

^1^ Departments of Nuclear Medicine, The First Affiliated Hospital of Xi’an Jiaotong University, Xi’an 710061, P.R. China

^2^ Medical Isotopes Research Center and Department of Radiation Medicine, School of Basic Medical Sciences, Peking University, Beijing 100191, P.R. China

^3^ Departments of Medical Oncology, The First Affiliated Hospital of Xi’an Jiaotong University, Xi’an 710061, P.R. China

^4^ Departments of General Surgery, The First Affiliated Hospital of Xi’an Jiaotong University, Xi’an 710061, P.R. China

^5^ Departments of Nuclear Medicine, Peking Union Medical College Hospital, Chinese Academy of Medical Sciences and Peking Union Medical College, Beijing 100730, P.R. China;

^6^ Foshan Atomical Medical Equipment Ltd.(S.C.), Tianfu Technology Center, Foshan 528000, P.R. China

**Address correspondence to**

Rui Gao (e-mail: [*jacky_mg@xjtufh.edu.cn*](mailto:jacky_mg@xjtufh.edu.cn)).

Departments of Nuclear Medicine, The First Affiliated Hospital of Xi’an Jiaotong University, No. 277 Yanta West Road, Xi’an 710061, P.R. China;

Tel: +86-137 7248 8039

OCRID: 0000-0003-4841-5929

^#^ Xi Jia, Xinru Li and Bing Jia contributed equally to this work.

**Supplementary methods**

**Eligibility criteria**

Inclusion criteria: (a) Subjects with newly diagnosed or previous treated esophageal cancer, cholangiocarcinoma, colorectal cancer, gastric cancer, pancreatic cancer, who intend to undergo pathological tissue biopsy or receive tumor surgical treatment in the near future (within 1 months); (b) patients who underwent ceCT to detect tumor recurrence and metastases (restaging); (c) The expected survival was more than 12 weeks; (d) Understand and sign informed consent voluntarily with good compliance.

Exclusion criteria: (a) Pathologically confirmed non-digestive system tumor; (b) The function of liver and kidney was seriously abnormal; (c) Preparation for pragnant, pregnant and lactating women; (d) Inability to lie flat for half an hour;(e) Suffering from claustrophobia or other mental disorders; (f) Other researchers considered it unsuitable to participate in the trial.

**Marterials and methods for synthesis of [^99m^Tc]Tc-HFAPi**

All reagents, unless otherwise specified, were of analytical grade and commercially available. High-quality Milli-Q water (18.2 MΩ/cm, Milipore, USA) was used for preparing solutions. [^99m^Tc]NaTcO_4_ was obtained from a commercial ^99^Mo/^99m^Tc generator (Beijing Atom High Tech Co., Ltd.). The radio high performance liquid chromatography (HPLC) method used a Hewlett Packard Series 1260 HPLC system equipped with Radioflow Detector LB509 and ReproSil-Pur Basic C18 column (10 mm × 250 mm, 5 μm particle size (Dr. Maisch, Germany)). The flow rate was 1 mL/min. The mobile phase was isocratic with 90% solvent A (Phosphate buffered saline (0.01 M PBS), pH 7.4) and 10% solvent B (acetonitrile) at 0 min, followed by a gradient mobile phase going from 10% solvent B to 40% solvent B at 17.5 min and to 10% solvent B at 20 min.

**ITLC method**

The radio-ITLC method used a thin layer chromatography Scanner AR-2000 (Bioscan, USA) and Gelman Sciences silica-gel paper strips in two different eluant. When 1:1 mixture of acetonitrile and saline as eluant, ^99m^Tc complexes and [^99m^Tc]TcO4^-^ migrated to the solvent front, while [^99m^Tc]Tc-colloid remained at the origin. When acetone as eluant, [^99m^Tc]TcO4^-^ migrated to the solvent front, while [^99m^Tc]Tc-colloid and ^99m^Tc complexes remained at the origin. The radiochemical purity was >95%. Since TPPTS is a reducing agent, no SnCl_2_ is needed. The [^99m^Tc]Tc-colloid was minimal (<0.5%) using the non-SnCl_2_ formulation.

**Supplementary figures**

**Fig. S1** Structure of [^99m^Tc]Tc-HFAPi

HYNIC, 6-hydrazinonicotinyl; TPPTS, trisodium triphenylphosphine-3,3′,3″-trisulfonate


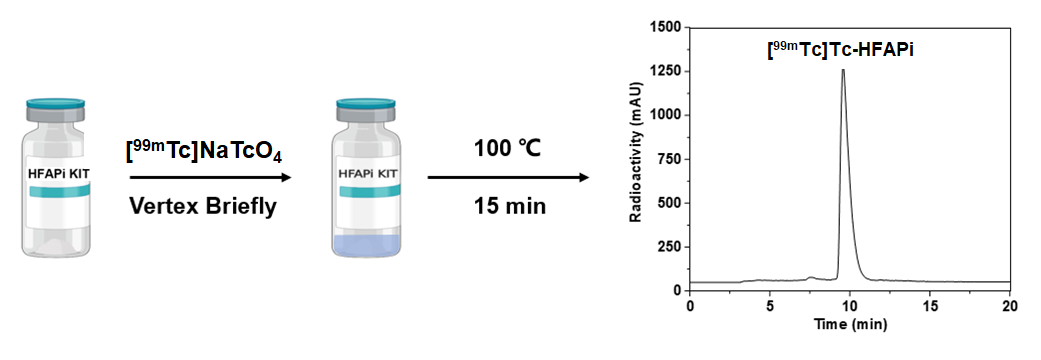


**Fig. S2** Preparation of [^99m^Tc]Tc-HFAPi by a kit formulation and the HPLC analysis of [^99m^Tc]Tc-HFAPi after radiolabeling.


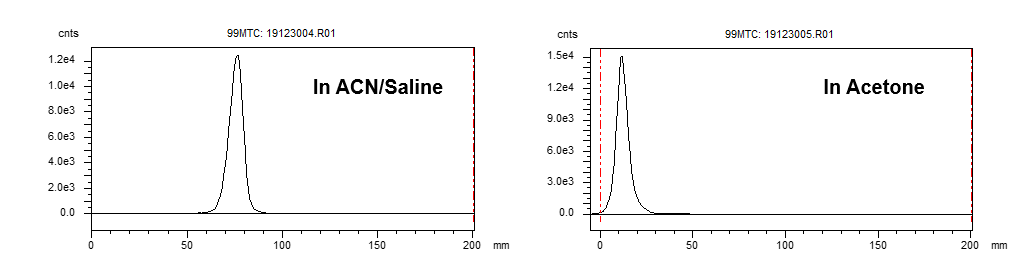


**Fig. S3** ITLC-SG analysis of [^99m^Tc]Tc-HFAPi after radiolabeling. (Left) 1:1 mixture of acetonitrile and saline as eluant. (Right) acetone as eluant.

**Supplementary tables**

**Table S1** Subject baseline characteristics and [^99m^Tc]Tc-HFAPi dose data

| No/Sex/Age(y) | Height (cm) | Weight (kg) | Injected Activity (MBq) |
| --- | --- | --- | --- |
| 1/M/76 | 165 | 60 | 895.4 |
| 2/M/60 | 176 | 78 | 913.9 |
| 3/F/51 | 156 | 47 | 832.5 |
| 4/F/72 | 150 | 41 | 832.5 |

**Table S2** Dosimetric data of [^99m^Tc]Tc-HFAPi

| Absorb dose of Target Organ  (mSv/MBq) | [^99m^Tc]Tc-HFAPi (n=4) | |
| --- | --- | --- |
|  | Mean | SD |
| Adrenals | 6.33E-04 | 8.03E-05 |
| Brain | 4.22E-04 | 3.81E-05 |
| Breasts | 2.36E-04 | 3.63E-05 |
| Gallbladder Wall | 5.69E-04 | 1.12E-04 |
| LLI Wall | 2.81E-05 | 2.69E-06 |
| Small Intestine | 1.18E-04 | 1.68E-05 |
| Stomach Wall | 4.04E-04 | 4.31E-05 |
| ULI Wall | 1.52E-04 | 2.53E-05 |
| Heart Wall | 4.27E-03 | 9.84E-04 |
| Kidneys | 2.75E-03 | 3.45E-04 |
| Liver | 1.45E-03 | 3.97E-04 |
| Lungs | 1.95E-03 | 3.25E-04 |
| Muscle | 1.45E-04 | 1.94E-05 |
| Ovaries | 4.06E-05 | 5.40E-06 |
| Pancreas | 4.24E-03 | 1.62E-04 |
| Red Marrow | 1.84E-04 | 2.31E-05 |
| Osteogenic Cells | 2.71E-04 | 3.29E-05 |
| Skin | 6.28E-05 | 8.28E-06 |
| Spleen | 1.21E-03 | 2.29E-04 |
| Testes | 4.28E-06 | 3.58E-07 |
| Thymus | 4.17E-04 | 7.42E-05 |
| Thyroid | 3.76E-03 | 1.11E-03 |
| Urinary Bladder Wall | 1.41E-05 | 1.56E-06 |
| Uterus | 3.63E-05 | 4.68E-06 |
| Total Body | 2.55E-04 | 3.47E-05 |
|  |  |  |
| Effective Dose Equivalent (mSv/MBq) | 1.26E-03 | 1.67E-04 |
| Effective Dose (mSv/MBq) | 6.80E-04 | 9.53E-05 |

**Table S3** Information of patients with postoperative recurrence

| No/Sex/Age(y) | Pathology | ceCT result | [^99m^Tc]Tc-HFAPi result (T/B ratio) | Ways to confirm |
| --- | --- | --- | --- | --- |
| 1/70/F | Colon adenocarcinoma | Positive | Positive (4.10) | Multi-modality medical imaging |
| 2/78/M | Anal malignant melanoma | Positive | Positive (2.55) | Multi-modality medical imaging |
| 3/57/M | Gastric adenocarcinoma | Positive | Positive (7.01) | Pathology confirm |

**Table S4** T/B ratio and SUV_max_ in different site of primary lesions by [^99m^Tc]Tc-HFAPi SPECT/CT

|  | Site | Stomach | Rectum | Colon | Others^a^ |
| --- | --- | --- | --- | --- | --- |
|  | (number) | (13) | (15) | (8) | (2) |
| T/B ratio | Median | 7.01 | 6.14 | 6.35 | / |
|  | IQR | 4.16-7.76 | 3.20-8.60 | 4.48-10.25 | 2.55-2.84 |
| SUV_max_ | Median | 12.43 | 7.96 | 9.13 | / |
|  | IQR | 5.81-15.80 | 4.03-9.98 | 6.10-13.57 | 2.45-3.70 |

^a^ esophageal cancer and anus melanoma

**Table S5** T/B ratio and SUV_max_ in different site of metastatic lesions by [^99m^Tc]Tc-HFAPi SPECT/CT

|  | Site | Liver | Bone | Peritoneum | Mediastinum | pelvic |
| --- | --- | --- | --- | --- | --- | --- |
|  | (number) | (17) | (8) | (1) | (1) | (1) |
| T/B ratio | median | 4.48 | 3.92 | 3.13 | 2.35 | 4.67 |
|  | IOR | 2.51-7.63 | 2.64-4.47 | / | / | / |
| SUV_max_ | median | 7.59 | 4.78 | 5.31 | 2.45 | 5.82 |
|  | IQR | 5.81-12.08 | 3.56-7.07 | / | / | / |
